# Supplementary material for: Analysis of Progression Time in Pancreatic Cancer including Carcinoma In Situ Based on Magnetic Resonance Cholangiopancreatography Findings
Source: Diagnostics (Basel). 2021 Oct 9;11(10):1858. doi: 10.3390/diagnostics11101858 (PMC8534569; doi:10.3390/diagnostics11101858)
Supplement: Supplementary file 1 [file diagnostics-11-01858-s001.zip › diagnostics-1351058-supplementary.pdf]

Supplementary Table S1. Diagnosis and first-time tumor detection in each patient

Patient No.	Final tumor size (mm)	Time to diagnosis (months)	Tumor size at first detection (mm)*	First-time tumor detection image modality*	Time to first detection (months)*
1	0†	23	–	–	–
2	0†	18	–	–	–
3	0†	26	–	–	–
4	3	50	4	CT	49
5	14	55	12	CT	54
6	16	26	8	CT	16
7	20	28	4	EUS	11
8	28	14	11	CT	11
9	36	39	7	EUS	16

* Based on the retrospective analysis performed by two independent reviewers. †CIS: Patients with CIS were treated as censored in the analysis of first tumor detection. Abbreviations: CIS, carcinoma in situ; CT, computed tomography; EUS, endoscopic ultrasound; MRI, magnetic resonance imaging.
